# Margarita (Pearl) Extract Alleviates Melasma by Targeting CAMP‐Responsive Element Binding Protein 1

**DOI:** 10.1111/jocd.70087

**Published:** 2025-05-14

**Authors:** Liling Shen, Jia Yao

**Affiliations:** ^1^ Department of Dermatology Zhejiang Provincial Dermatology Hospital Huzhou China

**Keywords:** CAMP‐responsive element binding protein 1, melasma, microphthalmia‐associated transcription factor, pearl extracts, tyrosinase

## Abstract

**Purpose:**

Margarita (pearl) has detoxifying and skin barrier‐repairing properties. The study was to evaluate the therapeutic effect of pearl on melasma and to preliminarily explore its possible mechanism of action.

**Methods:**

The efficacy of pearl on patients with melasma was evaluated by the melasma area and severity index (MASI) and physician's global assessment (PGA) score. DNA sequencing was performed on pharyngeal swab samples from patients with melasma to obtain gene loci related to melasma. The active compounds and potential targets for pearl were retrieved from Integrated Traditional Chinese Medicine and HERB databases. The relevant genes of melasma were obtained from the GeneCards database and intersected with the targets of pearl to identify potential targets of pearl against melasma. The potential targets were mapped for the KEGG pathway in the KEGG Mapper and verified using human melanoma A375 cells that were exposed to ultraviolet irradiation.

**Results:**

Pearl significantly improved the MASI and PGA scores of patients with melasma. DNA sequencing revealed that *TYR* and *DCT* were genes related to melasma. Calcium carbonate, iron, magnesium, manganese, silicon, strontium, and zinc were active compounds for pearl. CAMP‐responsive element binding protein 1 (CREB1) was the target of pearl against melasma. Pearl regulated microphthalmia‐associated transcription factors through CREB and affected melasma‐related genes *TYR* and *DCT*, which in turn inhibit melanoma cell activity and intracellular tyrosinase activity.

**Conclusion:**

Pearl can alleviate melasma by targeting the CREB1/MITF axis and then the melasma‐related gene loci *TYR* and *DCT*.

## 1 Introduction

Melasma (also known as chloasma) is a relatively common and acquired skin pigmentation disorder of sun‐exposed skin [1, 2]. It is characterized by symmetric patches of increased pigmentation in the epidermis and/or dermis, with irregular boundaries [3]. The prevalence of melasma is around 1% worldwide, with a higher prevalence (about 40%) in Southeast and South Asian populations [4]. Several factors, such as genetic susceptibility, sun exposure, and changes in sex hormone levels, contribute to melasma risk [5]. Increased melanin synthesis, vascular proliferation at the lesions, and inflammatory responses are involved in the development of melasma [6]. Microphthalmia‐associated transcription factor (MITF) is an important regulator of melanin formation and melanocyte development [7]. Several factors activate melanocyte function and promote melanin synthesis by acting directly or indirectly on MITF in melanocytes [8]. Melasma can be treated with a variety of therapeutic modalities, including systemic and topical pharmacological and procedural treatments. However, these treatments usually have a limited clinical response and are associated with unwanted adverse drug reactions [9]. Therefore, the development of safe and effective topical therapeutic agents is necessary in the management of melasma.

In Chinese medicine, it is believed that the onset of melasma is mainly related to the liver, kidney, and spleen. Traditional Chinese medicine, such as *extracts from Bletilla striata (Thunb.) Rchb. F., Centella asiatica (L.) Urb*., *Cynanchum atratum L., Rosa canina L., Rhus chinensis Mill*., *and Glycyrrhiza urolensis Fisch. Ex DC*., has been used as skin‐lightening agent [10]. Pearl has shown various effects, including osteogenic activity, antioxidant activity, anti‐inflammatory, and antiapoptotic effects [11]. It benefits from beauty care to healthcare, due to the active ingredients it contains [12]. The emerging biomedical applications of pearl have touched on the treatment of skin conditions [12]. Soluble pearl extract can lighten skin by antagonizing endothelin [13]. Pearl powder can reduce melanin content in the ultraviolet (UV)‐exposed skin, thus counteracting UV‐induced photoaging of skin [14]. However, considerable effort is required to unravel the potential mechanism of pearl against melasma.

The aim of this study was to evaluate the efficacy and potential targets of pearl extract in the treatment of melasma. The chemical composition of PE and its targets of action on melasma were predicted using network pharmacology. Since melanin deposition is the main cause of melasma, we established a model of UV‐induced A375 cells.

## 2 Material and Method

### 2.1 Extract Preparation

Pearls were sourced from the *Hyriopsis cumingii* . The pearl was ground using a stirring ball mill. Pearl powder was dissolved in lactic acid, using PBS to adjust the pH of the mixture to 7.0. Then, protease was added to initiate enzymatic hydrolysis. The extract was filtered. The filtrate was lyophilized and bottled for use.

### 2.2 Subjects

This is a single‐blind randomized trial (participants were unaware of the assignments). The study was approved by the Ethics Committee of Zhejiang Provincial Dermatology Hospital. Informed consent was obtained from all participants. Inclusion criteria: fulfilling the diagnostic criteria for melasma; female; aged 18–55 years; no history of any other medication or intervention before treatment; no other serious organ dysfunction; and with complete clinical data and informed consent. Exclusion criteria: patients unable to maintain strict sun protection during treatment; pregnant or breastfeeding women; individuals with mental abnormalities; and individuals unable to complete the treatment for other reasons. Eighty patients with melasma who were treated at the outpatient clinic of the hospital were selected for the study. The patients were randomly divided into two groups, each of which included 20 cases with epidermal‐type melasma and 20 cases with mixed‐type melasma. The melasma type was determined by Wood's light examination and confirmed by dermatoscope. The treatment for patients in the control group used hydroquinone cream (Qianbai, 10 g:0.2 g, Guangdong Renrenkang Pharmaceutical Co. Ltd., China), applied externally every night for 2 months. The participants in the PE group were treated with the above pearl extracts. Both treatments were applied externally every night for 2 months.

Improvement of patients was assessed using the melasma area and severity index (MASI) and physician's global assessment (PGA) scores, as described previously [15, 16].

### 2.3 Detection and Analysis of Genes Related to Melasma

Pharyngeal swab samples were collected from 80 patients with melasma. Sanger sequencing for single nucleotide polymorphisms and genomic association analysis was delegated to Youkang Biotechnology Co. Ltd. (Zhejiang, China) to find melanin‐related gene loci.

### 2.4 Network Pharmacology Analysis

The active ingredients of the pearls were obtained by searching the Integrated Traditional Chinese Medicine (http://itcm.biotcm.net/) and HERB (http://herb.ac.cn/) databases. The active ingredients of the pearls were entered into the Integrated Traditional Chinese Medicine database to predict their potential targets. The active ingredient–target network was constructed using Cytoscape.

GeneCards database was used to retrieve targets relevant to melasma. After harmonizing and standardizing, the common targets of the active ingredient with targets relevant to melasma were taken using Venny 2.1.0.

### 2.5 Cell Culture

A375 cells (SUNNCELL, Wuhan, China) were cultured in DMEM medium containing fetal bovine serum at 10% v/v. Cell growth conditions were 37°C and 5% CO_2_. Cells in the logarithmic growth phase were collected for subsequent experiments.

UV modeling and grouping: A375 cells were inoculated into 96‐well plates at 1 × 10^4^ cells/well and cultured for 24 h. Then, the cells were divided into four groups: control, UV, UV + PE, and UV + PE + forskolin (a CREB agonist). Except for the control group, the cells were exposed to UV irradiation at a dose of 30 J/cm^2^. The pearl extract was used at 1 g/mL. The cells in the UV + PE + pcDNA‐MITF group were pretransfected with MITF pcDNA (GenScript, China) using X‐treme GENE DNA transfection reagents (Roche, Germany).

### 2.6 Western Blot Analysis

Cells were lysed with cell lysis solution on ice for 15 min and centrifuged at 12000 rpm for 8 min at 4°C. Cell lysates were separated by SDS‐PAGE followed by Western blot analysis. The gels were stained with a vinylidene difluoride membrane and then exposed to the corresponding antibodies (pCREB, MITF, TYR, and DCT). Finally, after coupling the corresponding antibodies with horseradish peroxidase, the protein bands were visualized by ECL (DURASTAB, Shenzhen, China). Images were taken with a chemical imager and then analyzed on a gray scale using Image J software.

### 2.7 Cell Viability Analysis

Cell viability was assayed using the Cell Counting Kit‐8 (CCK8, SUNNCELL, China) assay. One‐hundred microliter of logarithmic growth phase A375 cells were inoculated at 1 × 10^5^ cells/mL in 96‐well plates and incubated for 24 h. After the addition of pearl extract to intervene in A375 cells for 4 h, the cells were irradiated with UV. Then, CCK8 (10 μL) was added for another incubation of 4 h. The absorbance value was measured at 450 nm using an enzyme meter.

### 2.8 Melanin Contents Analysis

A375 cells were cultured without/with pearl extract for 48 h. Then, the cells were washed with PBS solution, digested using trypsin, and centrifuged. The pellets were incubated for 2 h at 80°C in 1 M NaOH containing 10% DMSO. The absorbance (Abs) value was determined at 475 nm and expressed as Abs at 475 nm/10^5^ cells [17].

### 2.9 Intracellular Tyrosinase Activity Analysis

Tyrosinase activity in A375 cells was detected according to the method by Uto et al. [18]. In brief, A375 cells (1 × 105 cells/mL) were inoculated into cell culture plates and then incubated for 24 h. The cells were treated with pearl extract for 24 h. Treated cells were washed and lysed in 0.1 M sodium phosphate buffer (pH 6.8) containing 1% Triton X‐100 and a protease inhibitor mixture. The lysate was centrifuged, and the supernatant was collected. After protein concentration quantification using a dye‐binding protein assay kit (Bio‐Rad), 90 μL of equal amounts of protein lysate, along with 10 μL of 5 mM L‐DOPA, was added to the reaction mixture. After 2 h of incubation in the dark, the absorbance was measured at 415 nm using a microplate reader (iMark; Bio‐Rad). Tyrosinase activity was calculated from the absorbance ratio relative to that of the control culture.

### 2.10 Statistical Analysis

The data were expressed as mean ± SD. Unpaired *t*‐test, Chi‐square test, or one‐way analysis of variance was performed. *p* < 0.05 was considered statistically significant.

## 3 Results

### 3.1 Effect of PE Extract on Melasma Parameters MASI and PGA

**FIGURE 1:**
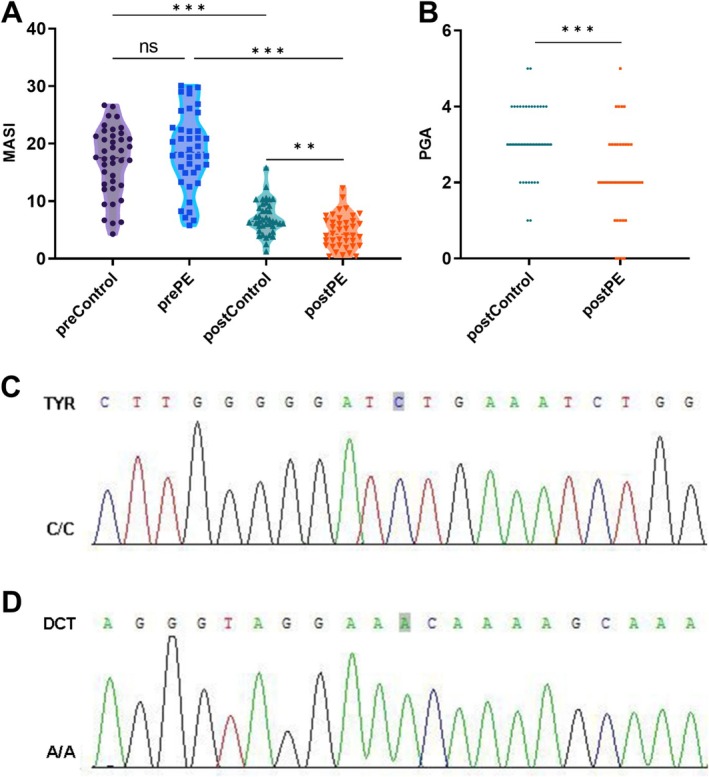
Effect of pearl extract on melasma and the detection of melasma‐related gene locus. (A) Melasma area and severity index were assessed pre‐ and posttreatment. (B) The physician's global assessment scores were recorded pre‐ and posttreatment. (C, D) Genotyping results of Sanger sequencing for gene loci TYR and DCT. **p < 0.01, ***p < 0.001.

The MASI scores of both the control and PE groups were significantly lower after treatment than those before treatment (*p* < 0.001, 95% CI: 7.136–12.82; Figure 1A). Nevertheless, the posttreatment reduction in MASI scores was more pronounced in the post‐PE group compared to the postcontrol group (*p* < 0.001, 95% CI: 9.436–15.12; Figure 1A). The PGA scores of the PE group were significantly better than those of the control group after treatment (Figure 1B). Representative pre‐ and posttreatment photos of a case with melasma are shown in Figure S1. Moreover, a 3‐month follow‐up showed that the MASI scores and PGA scores of the PE group were lower than those in the control group (Figure S2A,B). Sanger sequencing of pharyngeal swabs from 80 patients with melasma combined with genomic association analysis showed that TYR and DCT were the relevant genetic loci for melasma (Figure 1C,D).

### 3.2 PE Ingredient–Target Map

**FIGURE 2:**
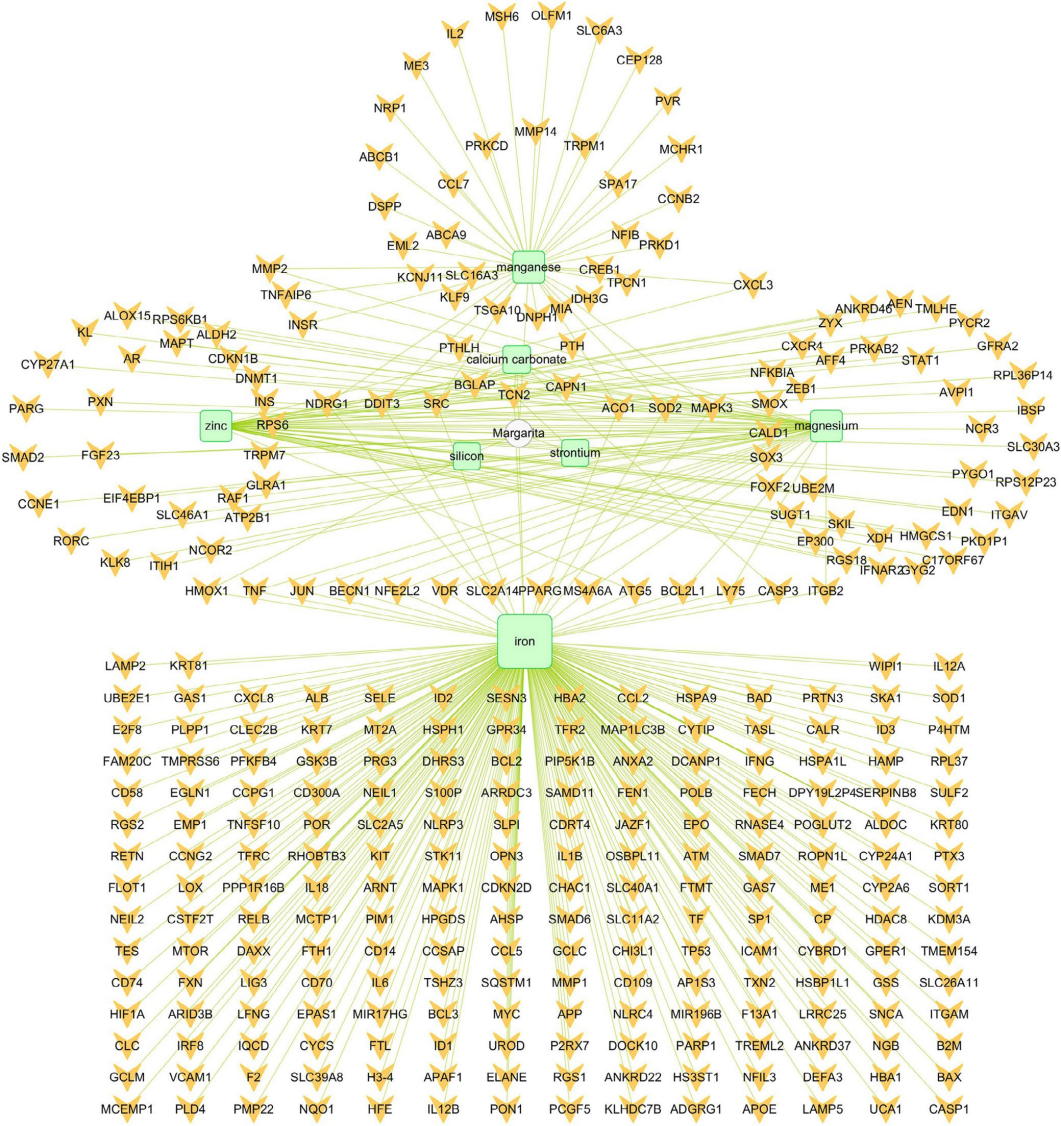
Network of 7 chemical compositions and 321 potential targets for margarita (pearl). Green round rectangle indicates chemical composition; Orange V indicates potential targets.

The Integrated Traditional Chinese Medicine and HERB databases proposed calcium carbonate, iron, magnesium, manganese, silicon, strontium, and zinc as active ingredients of pearls (Figure 2). These compounds targeted 321 potential targets, of which calcium carbonate targeted 7, iron targeted 221, magnesium targeted 40, manganese targeted 40, silicon targeted 1, strontium targeted 1, and zinc targeted 38 (Figure 2).

### 3.3 PE Affected Melasma‐Related Genes TYR and DCT Through CREB/MITF Axis

**FIGURE 3:**
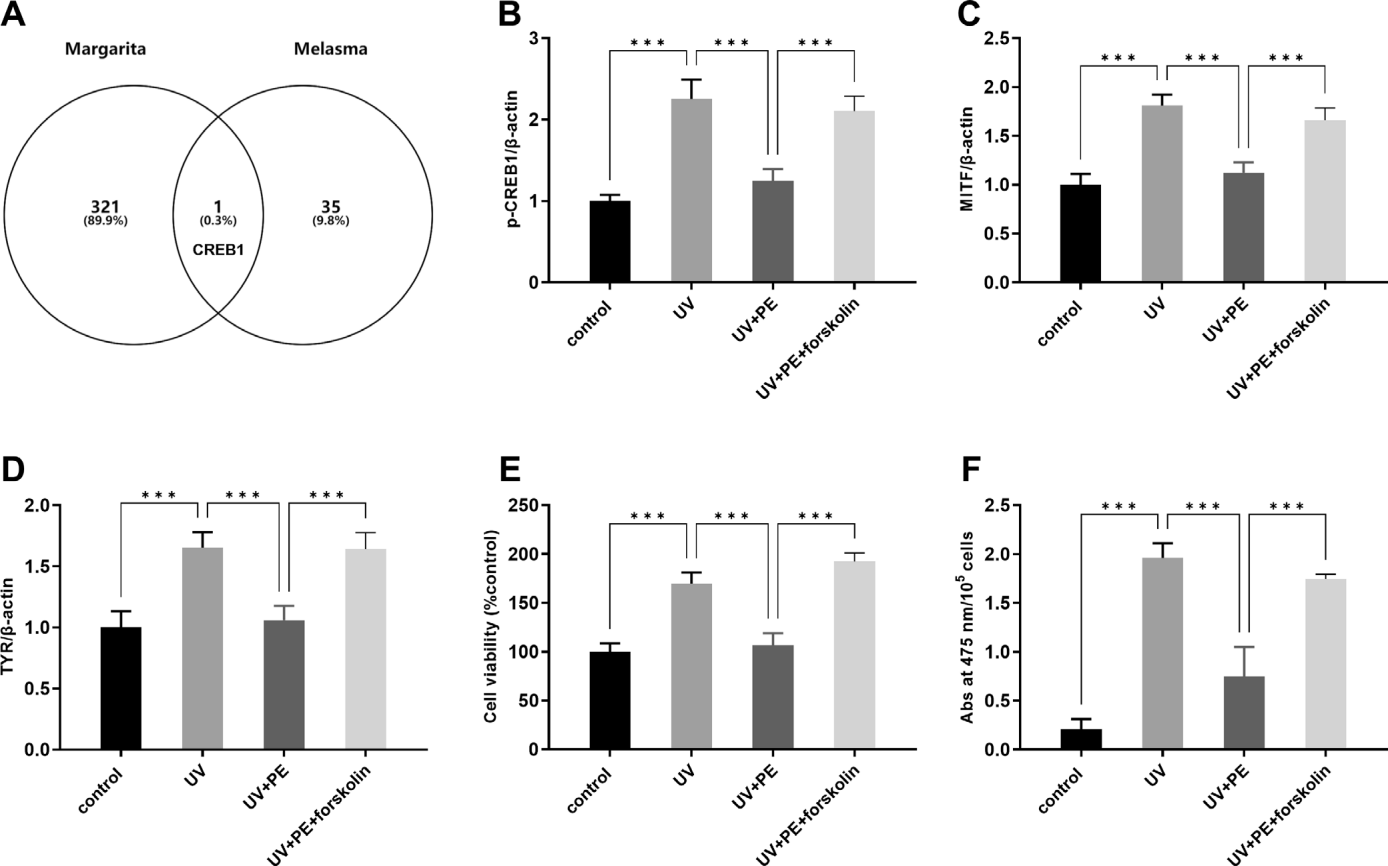
CREB1 was the potential target of pearl against melasma. (A) Venn diagram of predicted pearl‐related targets and melasma‐related genes from the GeneCards database. *p*‐CREB1 (B), MITF (C), and TYR (D) protein expression levels were detected by Western blot. (E) Cell viability was assayed using the CCK8 kit. (F) Melanin content was represented as absorbance (Abs) values at 475 nm. ***p < 0.001.

From the GeneCards database, a total of 36 genes related to melasma were obtained. The intersection of 322 (90.2%) targets of pearls and 36 (10.1%) targets of melasma disease yielded a total of 1 (0.3%) common target (CREB1) (Figure 3A). Interestingly, KEGG mapper showed a connection between CREB1 and melasma‐related genes TYR and DCT (Figure S3), with MITF as an intermediary. Western blot analysis showed that the protein expression of CREB1, TYR, and MITF was increased after UV exposure compared to the control group (Figure 3B–D). After the treatment of PE, the levels of all three proteins were significantly decreased compared to the UV group, which can be abolished by forskolin (Figure 3B–D). To further demonstrate that PE can be an effective treatment for melasma, we measured melanocyte viability and melanin content. The results showed that cell viability and Abs at 475 nm/10^5^ cells were significantly higher in the UV group compared to the control group (Figure 3E,F). However, cell viability and Abs at 475 nm/10^5^ cells were reduced after pearl treatment, though forskolin can offset these effects (Figure 3E,F).

### 3.4 MITF Overexpression Reverses the Role of PE

**FIGURE 4:**
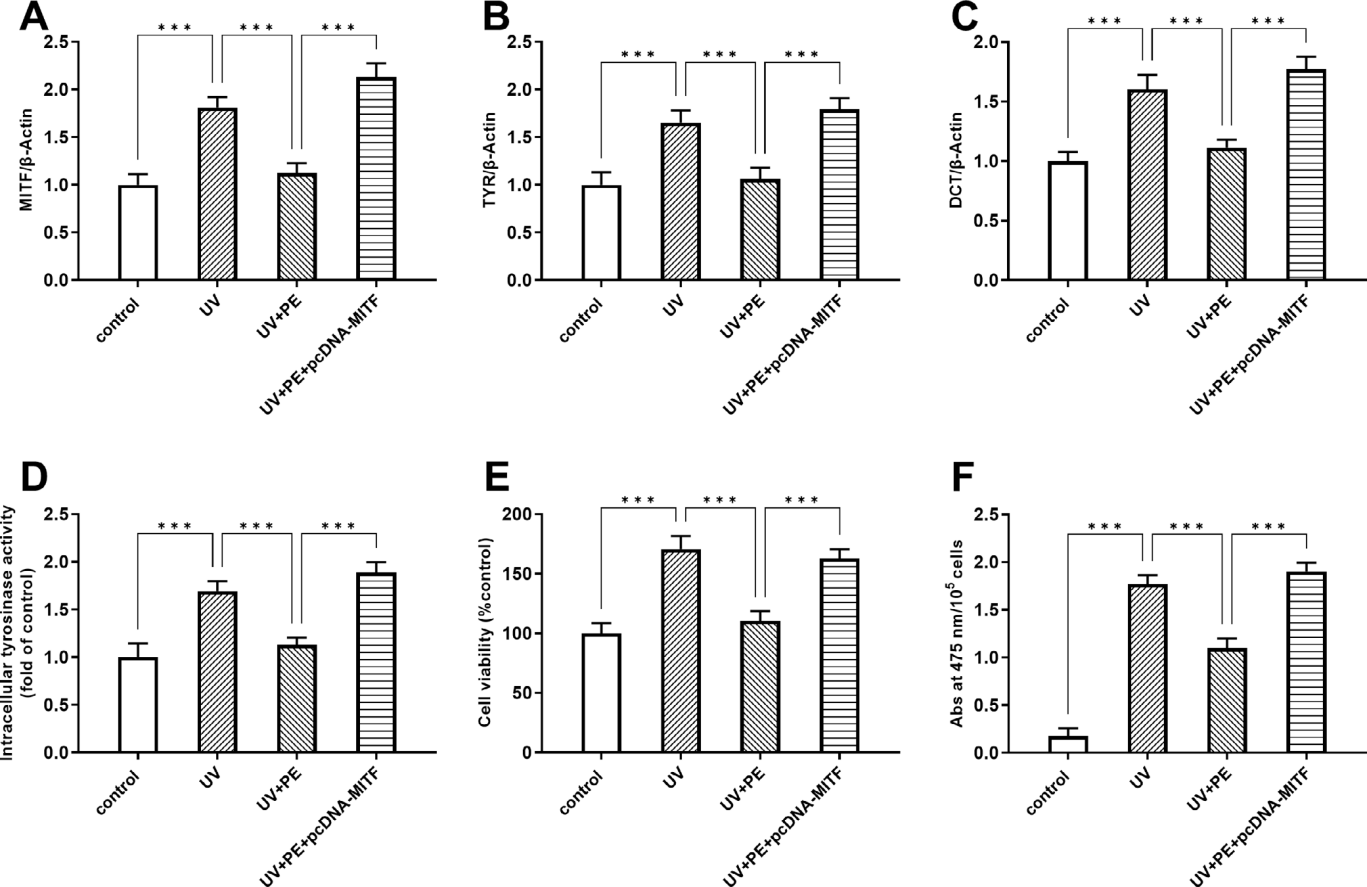
MITF overexpression reversed the role of PE. MITF (A), TYR (B), and DCT (C) protein expression levels were detected by Western blot with/without MITF overexpression. (D) Intracellular tyrosinase activity was determined with/without MITF overexpression. (E) A375 cell viability was assayed using the CCK8 kit with/without MITF overexpression. (F) Melanin content was detected by photometry at 415 nm. ***p < 0.001.

MITF is a major regulator of melanogenesis, and it is the ultimate target of multiple signaling pathways [19]. We then further validated the CREB/MITF axis in PE against melasma using MITF overexpression (Figure 4A). TYR and DCT levels in UV‐exposed/PE‐treated cells were increased after MITF overexpression (Figure 4B,C). Since tyrosinase is a key enzyme in melanin formation, we examined intracellular tyrosinase activity. The results showed that intracellular tyrosinase activity in UV‐exposed/PE‐treated cells was increased after MITF overexpression (Figure 4D). Similarly, melanocyte viability and melanin content (Abs at 475 nm/10^5^ cells) in UV‐exposed/PE‐treated cells were significantly higher after MITF overexpression (Figure 4E,F).

## 4 Discussion

This study revealed the possible mechanism of PE in the treatment of melasma with the help of precise genetic testing technology and cell experiments. Our results showed that PE extract can confront melasma, improving patients' skin status. By constructing a network pharmacological model, the potential targets of PE were analyzed. The potential mechanism of PE extract against melasma was predicted to target the CREB1/MITF/TYR/DCT axis. It was demonstrated that PE can regulate MITF through CREB, which then affects melasma‐related genes TYR and DCT.

Pearls have been used for skin care in China for over a thousand years [12]. It has been shown to have antioxidant and antiradiation properties [11]. Pearl powder at a nanoscale degree can accelerate wound repair and regeneration [20]. Pearl powder contains several trace elements and active proteins that can nourish the skin. Using network pharmacology, calcium carbonate, iron, magnesium, manganese, silicon, strontium, and zinc were proposed to be active ingredients in pearl in this study. Calcium carbonate can act as a carrier for the therapy of melanoma [21]. The presence of an excessive amount of iron can make melanin to be prooxidant [22]. Iron can bind to oxidatively modified melanin, participating in the degradation of melanin [22]. Pigmentations are largely determined by the ability of melanin biopolymers to bind various metal ions and chemicals such as drugs [23]. Magnesium can decrease the number of total binding sites in aminoglycoside–melanin complexes [24]. Silica combined with zinc oxide nanoparticles can reduce the potential toxicity and produce photoprotective effects for skin against hyperpigmentation [25]. Our results showed that pearl extract can improve the MASI and PGA scores of patients with melasma significantly, indicating that pearls can improve the skin condition of patients with melasma.

Melanogenesis is a complex genetic and epigenetic process involving multiple genes and signaling pathways [26]. The rapid development of gene technology has led to control of chronic and genetic diseases in human beings, including skin disease [27]. In this study, Sanger sequencing of pharyngeal swabs from melasma populations and genomic association analysis revealed that TYR and DCT were the relevant genetic loci for melasma. It has been shown that the TYR gene is duplicated and mutated to eventually form TYR‐1 and DCT, which are directly involved in the process of melanogenesis [28, 29]. MITF can make melanocytes resistant to UV‐induced apoptosis due to its activation of the target genes TYR and DCT [30]. Here, we found that MITF overexpression can reverse the effects of pearl in melasma, suggesting the involvement of the MITF/TYR/DCT axis in the antimelasma mechanism of pearl.

**FIGURE 5:**
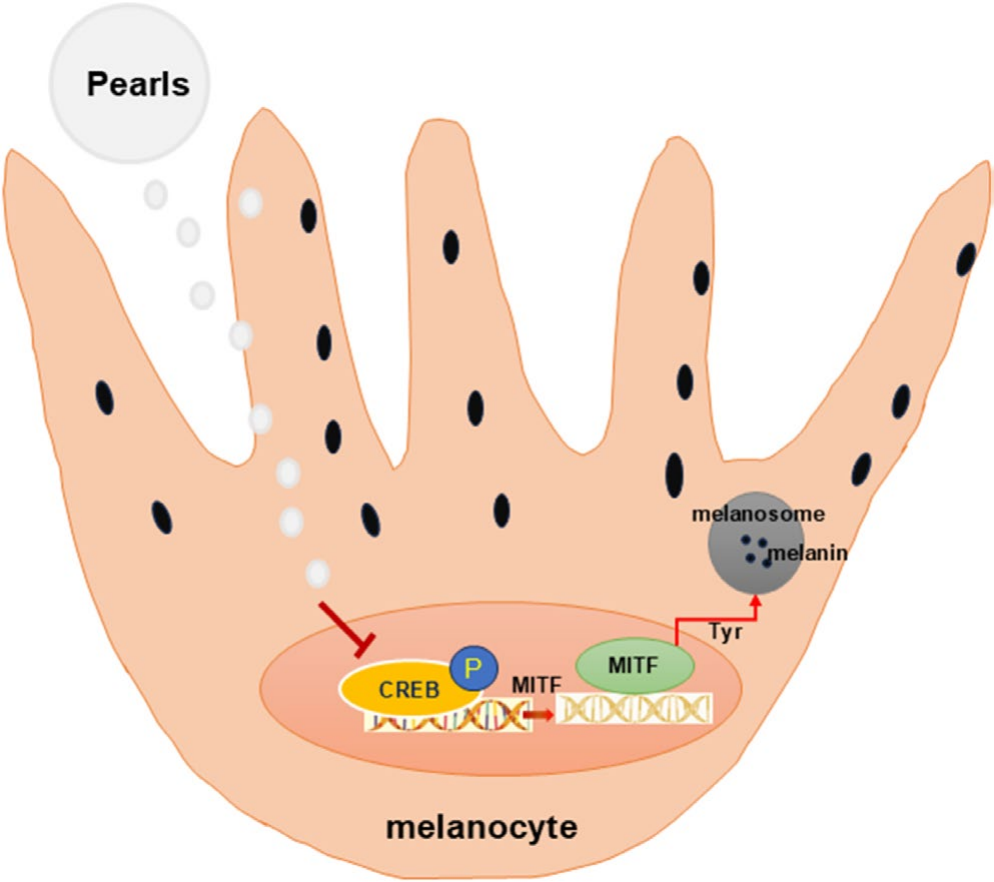
The potential mechanism of PE in treating melanoma.

Network pharmacology is from a cyber perspective, to elucidate the effect mechanism of the multimolecular synergistic action of herbal medicine constituents [31]. In this study, we utilized network pharmacology to predict the potential targets of pearl in melasma. We found CREB1 was a potential target of pearl in melasma. It has been shown that melanocyte‐stimulating hormone (MSH) can transcribe the MITF gene by upregulating CREB expression. MITF in turn leads to an increase in tyrosinase expression, which in turn catalyzes the breakdown of tyrosine, leading to an increase in melanin levels [32]. Our results revealed that PE can inhibit melanoma cell viability and melanin content. Therefore, pearl extract can confront melasma by targeting CREB1, thus regulating the MITF/TYR/DCT axis (Figure 5).

One of the main shortcomings of this study was the fact that no other genders, apart from females, were included in the analyses. Additionally, we did not conduct in‐depth research on the therapeutic differences of pearls in different types of melasma. Furthermore, in vivo tests or long‐term studies are needed for the curative effect of pearl in melasma. The active components in PE that do the heavy lifting should be identified. All these would be our future work focus.

## 5 Conclusion

This study showed that PE may target CREB1 and thus regulate the MITF/TYR/DCT axis to reduce melanoma cell viability and the production of melanin. However, the active ingredients and mechanism of action of PE still need to be further elucidated.

## Author Contributions

Conceptualization: L.S. and J.Y.; methodology: L.S. and J.Y.; investigation: L.S. and J.Y.; data analysis: L.S. and J.Y.; writing – original draft preparation: L.S.; and writing – review and editing: J.Y.

## Ethics Statement

The study was approved by the Ethics Committee of Zhejiang Provincial Dermatology Hospital. Informed consent was obtained from all participants.

## Conflicts of Interest

The authors declare no conflicts of interest.

## Supporting information


**Figure S1.** Representative pre‐ and posttreatment photos of a case with melasma.

**Figure S2.** The MASI scores (A) and PGA scores (B) of the two groups in the 3‐month follow‐up.

**Figure S3.** The involved genes in the melanogenesis signaling pathway (from KEGG Mapper).

## Data Availability

The data that support the findings of this study are available from the corresponding author upon reasonable request.

## References

[1] A. C. C. Espósito , G. Brianezi , N. P. de Souza , L. D. B. Miot , and H. A. Miot , “Exploratory Study of Epidermis, Basement Membrane Zone, Upper Dermis Alterations and Wnt Pathway Activation in Melasma Compared to Adjacent and Retroauricular Skin,” Annals of Dermatology 32, no. 2 (2020): 101–108, 10.5021/ad.2020.32.2.101. 33911720 PMC7992552

[2] O. A. Ogbechie‐Godec and N. Elbuluk , “Melasma: An up‐To‐Date Comprehensive Review,” Dermatology and Therapy 7, no. 3 (2017): 305–318, 10.1007/s13555-017-0194-1. 28726212 PMC5574745

[3] F. Qu , R. Geng , Y. Liu , and J. Zhu , “Advanced Nanocarrier‐ and Microneedle‐Based Transdermal Drug Delivery Strategies for Skin Diseases Treatment,” Theranostics 12, no. 7 (2022): 3372–3406, 10.7150/thno.69999. 35547773 PMC9065205

[4] B. Doolan and M. Gupta , “Melasma,” Australian Journal for General Practitioners 50 (2021): 880–885. 10.31128/AJGP-05-21-6002 34845463

[5] S. Rajanala , M. B. C. Maymone , and N. A. Vashi , “Melasma Pathogenesis: A Review of the Latest Research, Pathological Findings, and Investigational Therapies,” Dermatology Online Journal 25, no. 10 (2019): 13030/qt47b7r2, 10.5070/d32510045810. 31735001

[6] A. C. C. Espósito , D. P. Cassiano , C. N. da Silva , et al., “Update on Melasma‐Part I: Pathogenesis,” Dermatology and Therapy 12, no. 9 (2022): 1967–1988, 10.1007/s13555-022-00779-x. 35904706 PMC9464278

[7] M. C. Gelmi , R. M. Verdijk , L. E. Houtzagers , et al., “Microphthalmia‐Associated Transcription Factor: A Differentiation Marker in Uveal Melanoma,” International Journal of Molecular Sciences 24, no. 10 (2023): 8861, 10.3390/ijms24108861. 37240204 PMC10218684

[8] T. Pillaiyar , M. Manickam , and S. H. Jung , “Downregulation of Melanogenesis: Drug Discovery and Therapeutic Options,” Drug Discovery Today 22, no. 2 (2017): 282–298, 10.1016/j.drudis.2016.09.016. 27693716

[9] P. Ghasemiyeh , R. Fazlinejad , M. R. Kiafar , S. Rasekh , M. Mokhtarzadegan , and S. Mohammadi‐Samani , “Different Therapeutic Approaches in Melasma: Advances and Limitations,” Frontiers in Pharmacology 15 (2024): 1337282, 10.3389/fphar.2024.1337282. 38628650 PMC11019021

[10] T. Liu , Y. Lu , K. Tonissen , G. Di Trapani , W. Tang , and Y. Feng , “Application of Traditional Chinese Medicine as Skin Depigmentation Agents,” Heliyon 8, no. 12 (2022): e12571, 10.1016/j.heliyon.2022.e12571. 36636217 PMC9830152

[11] J. Pei , Y. Wang , X. Zou , et al., “Extraction, Purification, Bioactivities and Application of Matrix Proteins From Pearl Powder and Nacre Powder: A Review,” Frontiers in Bioengineering and Biotechnology 9 (2021): 649665, 10.3389/fbioe.2021.649665. 33959598 PMC8095667

[12] X. J. Loh , D. J. Young , H. Guo , et al., “Pearl Powder‐An Emerging Material for Biomedical Applications: A Review,” Materials 14, no. 11 (2021): 2797, 10.3390/ma14112797. 34074019 PMC8197316

[13] J. Wang , Z. Chen , Y. Lu , et al., “Soluble Pearl Extract Provides Effective Skin Lightening by Antagonizing Endothelin,” Journal of Cosmetic Dermatology 20, no. 8 (2021): 2531–2537, 10.1111/jocd.13899. 33355986

[14] S. Han , D. Huang , T. Lan , et al., “Therapeutic Effect of Seawater Pearl Powder on UV‐Induced Photoaging in Mouse Skin,” Evidence‐Based Complementary and Alternative Medicine: Ecam 2021 (2021): 9516427, 10.1155/2021/9516427. 34925534 PMC8677389

[15] A. Tourlaki , M. G. Galimberti , G. Pellacani , and P. L. Bencini , “Combination of Fractional Erbium‐Glass Laser and Topical Therapy in Melasma Resistant to Triple‐Combination Cream,” Journal of Dermatological Treatment 25, no. 3 (2014): 218–222, 10.3109/09546634.2012.671911. 22385073

[16] S. Y. Salma , S. Nikhat , M. Manjhi , M. W. Akhtar , and S. Ahmad , “Clinical Evaluation of a Topical Unani Pharmacopoeial Formulation Tila‐e‐Kalf in the Management of Melasma (Kalf): A Randomized Controlled Clinical Trial,” Avicenna Journal of Phytomedicine 13, no. 3 (2023): 255–264, 10.22038/ajp.2022.21346. 37654999 PMC10465884

[17] S. Huh , E. Jung , J. Lee , et al., “Mechanisms of Melanogenesis Inhibition by Propafenone,” Archives of Dermatological Research 302, no. 7 (2010): 561–565, 10.1007/s00403-010-1059-y. 20549222

[18] T. Uto , T. Ohta , K. Katayama , and Y. Shoyama , “Silibinin Promotes Melanogenesis Through the PKA and p38 MAPK Signaling Pathways in Melanoma Cells,” Biomedical Research 43, no. 2 (2022): 31–39, 10.2220/biomedres.43.31. 35431290

[19] S. Kumari , S. Tien Guan Thng , N. Kumar Verma , and H. K. Gautam , “Melanogenesis Inhibitors,” Acta Dermato‐Venereologica 98, no. 10 (2018): 924–931, 10.2340/00015555-3002. 29972222

[20] X. Chen , L. H. Peng , S. S. Chee , Y. H. Shan , W. Q. Liang , and J. Q. Gao , “Nanoscaled Pearl Powder Accelerates Wound Repair and Regeneration In Vitro and In Vivo,” Drug Development and Industrial Pharmacy 45, no. 6 (2019): 1009–1016, 10.1080/03639045.2019.1593436. 30950303

[21] T. E. Karpov , A. Rogova , D. R. Akhmetova , et al., “Encapsulation of a Small‐Molecule Drug Based on Substituted 2‐Aminothiophenes in Calcium Carbonate Carriers for Therapy of Melanoma,” Biomaterials Science 12, no. 13 (2024): 3431–3445, 10.1039/d4bm00390j. 38812410

[22] A. C. Żądło and T. Sarna , “Interaction of Iron Ions With Melanin,” Acta Biochimica Polonica 66, no. 4 (2019): 459–462, 10.18388/abp.2019_2889. 31826048

[23] R. M. Slominski , T. Sarna , P. M. Płonka , C. Raman , A. A. Brożyna , and A. T. Slominski , “Melanoma, Melanin, and Melanogenesis: The Yin and Yang Relationship,” Frontiers in Oncology 12 (2022): 842496, 10.3389/fonc.2022.842496. 35359389 PMC8963986

[24] D. Wrześniok , E. Buszman , and E. Miernik‐Biela , “Amikacin, Kanamycin and Tobramycin Binding to Melanin in the Presence of ca(2+) and mg(2+) Ions,” Acta Poloniae Pharmaceutica 69, no. 6 (2012): 1035–1041. 23285663

[25] S. M. Kandil , H. M. Diab , A. M. Mahfoz , A. Elhawatky , and E. M. Abdou , “Duo Photoprotective Effect via Silica‐Coated Zinc Oxide Nanoparticles and Vitamin C Nanovesicles Composites,” Pharmaceutical Research 41, no. 7 (2024): 1475–1491, 10.1007/s11095-024-03733-y. 38992234 PMC11263436

[26] S. Zhou , H. Zeng , J. Huang , et al., “Epigenetic Regulation of Melanogenesis,” Ageing Research Reviews 69 (2021): 101349, 10.1016/j.arr.2021.101349. 33984527

[27] A. Schäbitz , C. Hillig , M. Mubarak , et al., “Spatial Transcriptomics Landscape of Lesions From Non‐Communicable Inflammatory Skin Diseases,” Nature Communications 13, no. 1 (2022): 7729, 10.1038/s41467-022-35319-w. PMC9747967 36513651

[28] C. Jiménez‐Cervantes , F. Solano , T. Kobayashi , et al., “A New Enzymatic Function in the Melanogenic Pathway. The 5,6‐Dihydroxyindole‐2‐Carboxylic Acid Oxidase Activity of Tyrosinase‐Related Protein‐1 (TRP1),” Journal of Biological Chemistry 269, no. 27 (1994): 17993–18000. 8027058

[29] J. S. Yu and A. K. Kim , “Effect of Combination of Taurine and Azelaic Acid on Antimelanogenesis in Murine Melanoma Cells,” Journal of Biomedical Science 17, no. 1 (2010): 45, 10.1186/1423-0127-17-s1-s45. 20804622 PMC2994384

[30] T. J. Hornyak , S. Jiang , E. A. Guzmán , et al., “Mitf Dosage as a Primary Determinant of Melanocyte Survival After Ultraviolet Irradiation,” Pigment Cell & Melanoma Research 22, no. 3 (2009): 307–318, 10.1111/j.1755-148X.2009.00551.x. 19192212 PMC6980044

[31] R. Zhang , X. Zhu , H. Bai , and K. Ning , “Network Pharmacology Databases for Traditional Chinese Medicine: Review and Assessment,” Frontiers in Pharmacology 10 (2019): 123, 10.3389/fphar.2019.00123. 30846939 PMC6393382

[32] M. Bouhoute , Y. Amen , M. Bejaoui , M. AKO , K. Shimizu , and H. Isoda , “New Butyroside D From Argan Press Cake Possess Anti‐Melanogenesis Effect via MITF Downregulation in B16F10 and HEM Cells,” International Journal of Molecular Sciences 23 (2022): 16021, 10.3390/ijms232416021202216021. 36555664 PMC9785346

