# Supplementary figures and images for: Margarita (Pearl) Extract Alleviates Melasma by Targeting CAMP‐Responsive Element Binding Protein 1
Source: J Cosmet Dermatol. 2025 May 14;24(5):e70087. doi: 10.1111/jocd.70087 (PMC12079008; doi:10.1111/jocd.70087)

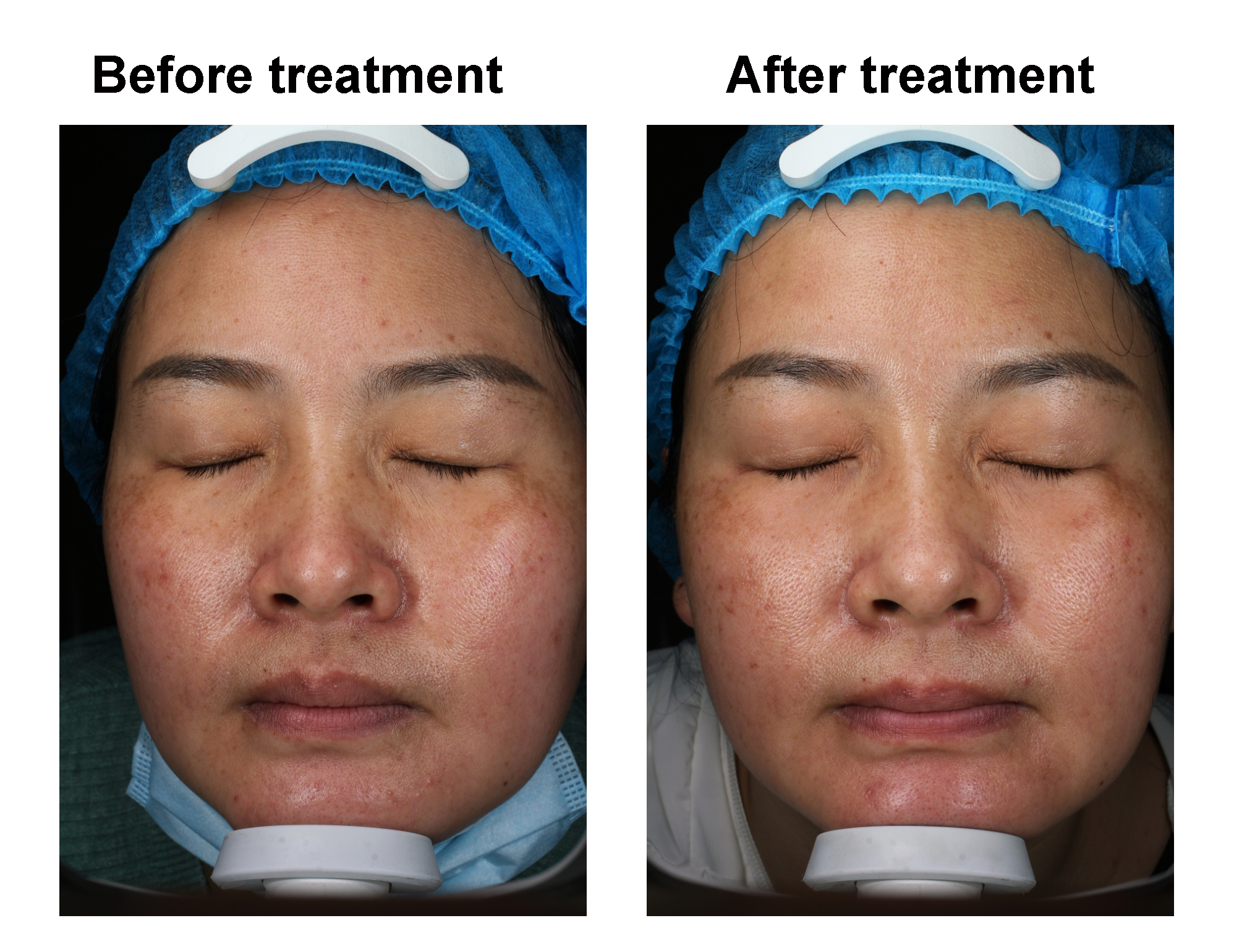

Supplement: Supplementary file 1 — Figure S1. Representative pre‐ and posttreatment photos of a case with melasma. [file JOCD-24-e70087-s001.tif]

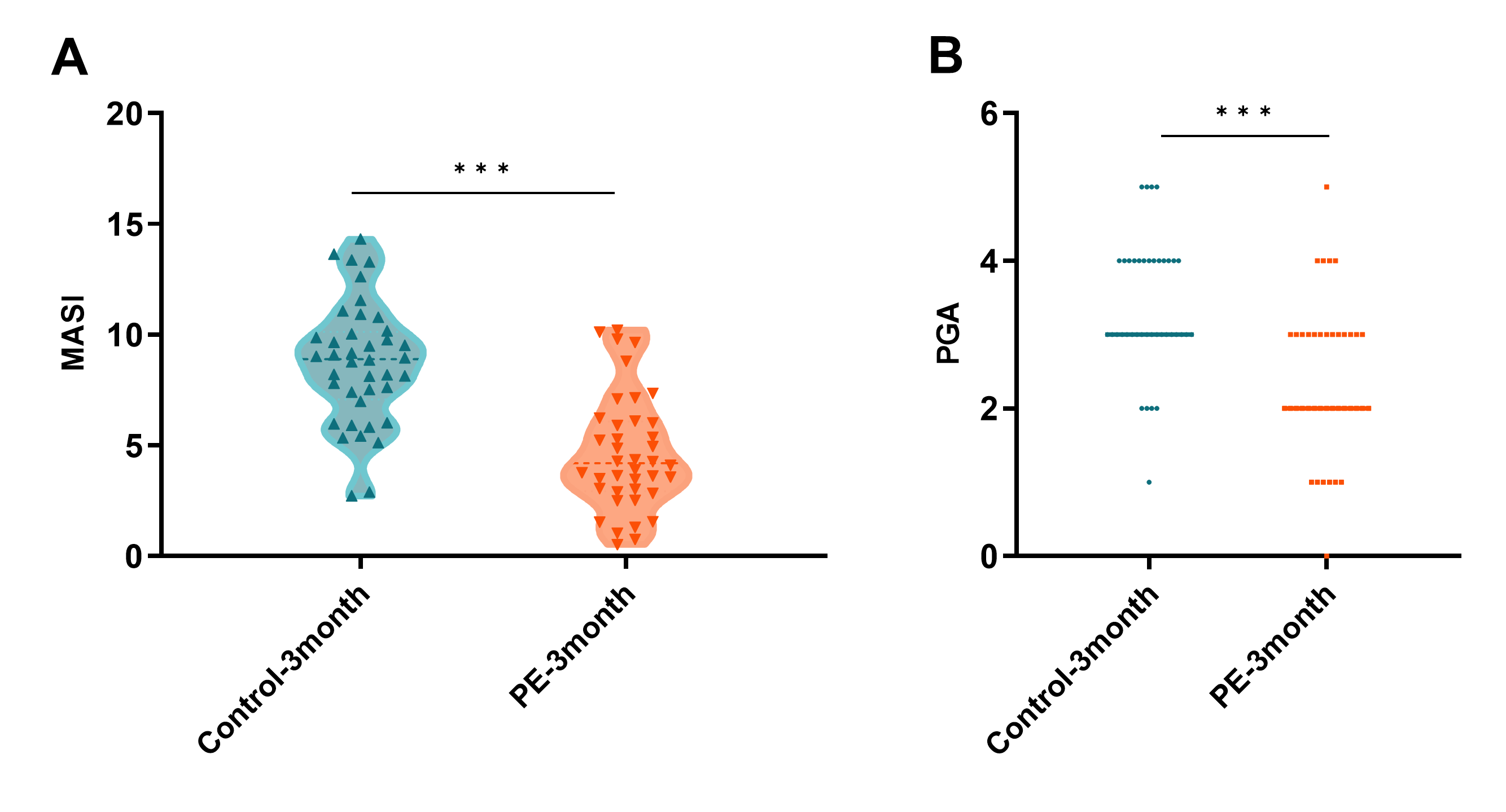

Supplement: Supplementary file 2 — Figure S2. The MASI scores (A) and PGA scores (B) of the two groups in the 3‐month follow‐up. [file JOCD-24-e70087-s003.tif]

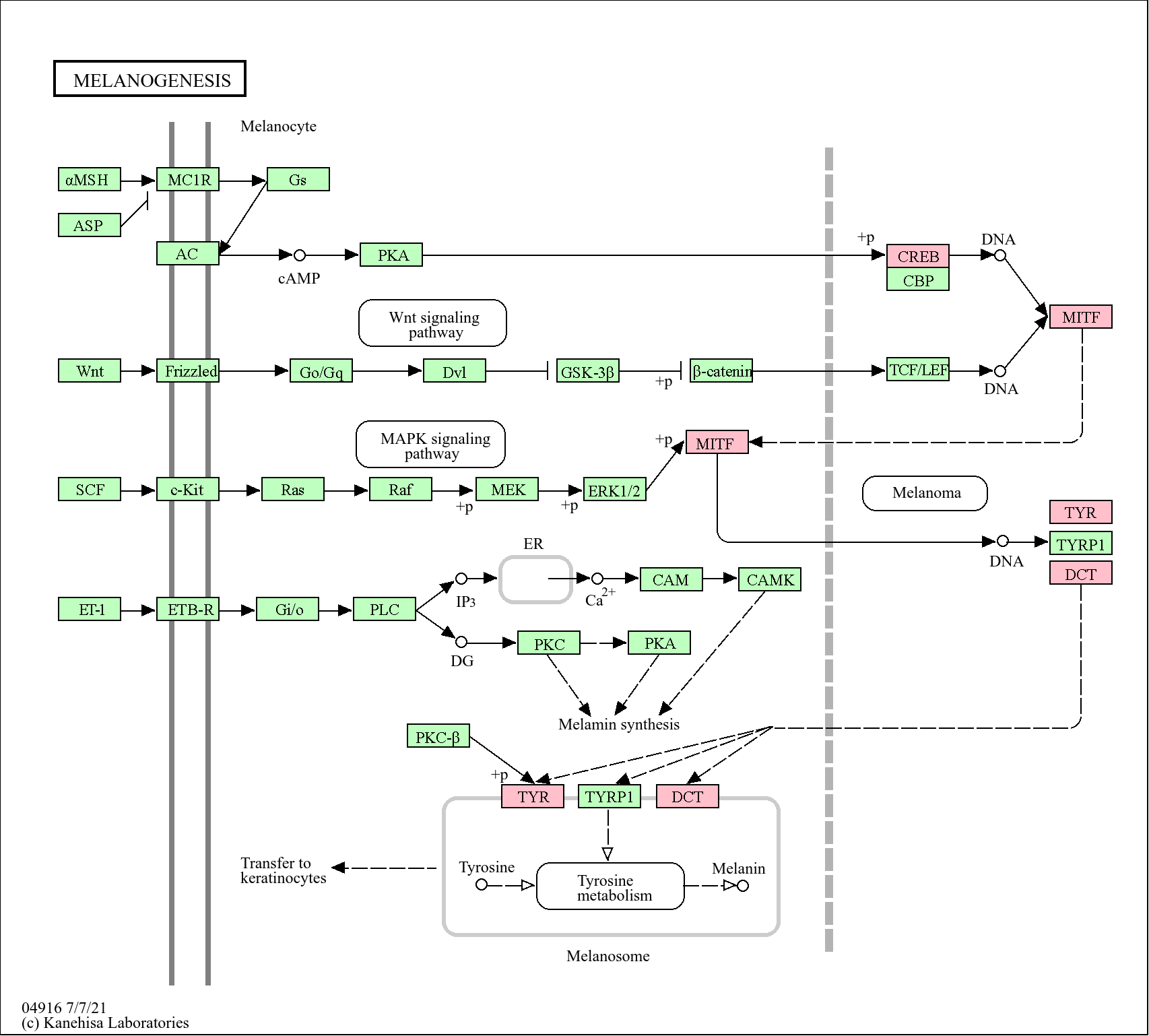

Supplement: Supplementary file 3 — Figure S3. The involved genes in the melanogenesis signaling pathway (from KEGG Mapper). [file JOCD-24-e70087-s002.png]
